# Protocol for iterative optimization of modified peptides bound to protein targets

**DOI:** 10.1007/s10822-022-00482-1

**Published:** 2022-10-19

**Authors:** Rodrigo Ochoa, Pilar Cossio, Thomas Fox

**Affiliations:** 1 Biophysics of Tropical Diseases, Max Planck Tandem Group, University of Antioquia, Medellín, 050010 Colombia; 2 Medicinal Chemistry, Boehringer Ingelheim Pharma GmbH & Co KG, 88397 Biberach/Riss, Germany; 3 Center for Computational Mathematics, Flatiron Institute, New York, 10010 USA; 4 Center for Computational Biology, Flatiron Institute, New York, 10010 USA

**Keywords:** Peptide design, Computational chemistry, Non-natural amino acids, Monte Carlo

## Abstract

Peptides are commonly used as therapeutic agents. However, they suffer from easy degradation and instability. Replacing natural by non-natural amino acids can avoid these problems, and potentially improve the affinity towards the target protein. Here, we present a computational pipeline to optimize peptides based on adding non-natural amino acids while improving their binding affinity. The workflow is an iterative computational evolution algorithm, inspired by the PARCE protocol, that performs single-point mutations on the peptide sequence using modules from the Rosetta framework. The modifications can be guided based on the structural properties or previous knowledge of the biological system. At each mutation step, the affinity to the protein is estimated by sampling the complex conformations and applying a consensus metric using various open protein-ligand scoring functions. The mutations are accepted based on the score differences, allowing for an iterative optimization of the initial peptide. The sampling/scoring scheme was benchmarked with a set of protein-peptide complexes where experimental affinity values have been reported. In addition, a basic application using a known protein-peptide complex is also provided. The structure- and dynamic-based approach allows users to optimize bound peptides, with the option to personalize the code for further applications. The protocol, called mPARCE, is available at: https://github.com/rochoa85/mPARCE/.

**Supplementary Information:**

The online version contains supplementary material available at 10.1007/s10822-022-00482-1.

## Introduction

The use of peptides for biomedical and biotechnological purposes has several advantages, including potential lower adverse effects due to their extremely high affinity and specificity during the binding events [1]. However, they are associated with poor chemical and physical stability, short circulating plasma half-life, and proteolysis [2, 3]. Moreover, they can be easily cleaved by proteases. This has motivated the design of modified peptides, which contain at least one non-natural amino acid (NNAA) [4]. These changes can protect the molecule of being cleaved due to chemical modifications on their side or main chains [5, 6]. In general, natural and modified peptides can be designed by computational protocols able to improve observables such as affinity scores towards a protein target, or certain physico-chemical properties like hydrophobicity profiles [7, 8].

Among the computational design strategies, there are methodologies relying on molecular simulations for studying potential binding, and subsequently suggesting mutations on the peptide that can potentially improve their affinity [9]. This is the case of PARCE, a method to optimize natural peptide-binders, where it has been shown that conformational sampling and an efficient exploration of the sequence space are necessary [10]. Another example was the design of peptides that bind small organic molecules by taking into account different solvents in the simulations [11]. The approach was based on a Monte Carlo search in the space of possible peptides, simulated with finite temperature molecular dynamics (MD) [12, 13]. These hybrid computational strategies have been applied in the past for the design of MHC II peptide binders [14–16], and the engineering of nanobodies [17–19] by combining explicit solvent MD configurations, or Monte Carlo generated trajectories, with consensus scoring approaches, which can efficiently assess the impact on binding given a mutation on the peptide binder. There are other reported methodologies to design peptides by extracting information from protein-protein interfaces [20, 21], or by using hyperstable backbone conformations to fit designed peptide sequences [22].

In the case of peptides modified with NNAAs, there are methodologies able to model and parameterize the monomers [23, 24] to include them for the computational binding estimations [25, 26]. The conformational sampling can be explored by MD simulations or more computationally efficient e.g., Monte Carlo movers. Some of these methods are found in the Rosetta Commons project [27], which include efficient flexible-backbone sampling to investigate the interactions in the complex [24, 28], like the Backrub approach [29]. These methods can incorporate biological restraints to optimize the exploration based on previous knowledge of the system [30]. However, a limitation is the prediction of affinity scores between the peptide and the target that is still challenging given the high flexibility of the peptides and the lack of specific force-fields for NNAAs.

In our method, we implement a consensus approach supported by the Rosetta framework able to overcome some of the aforementioned challenges. We developed a protocol based on PARCE to design modified peptides with improved binding affinity to a target. The protocol, called mPARCE, generates single-point mutations on the peptide sequence based on a list of parameterized *α*- L- and D-NNAAs. Then, it estimates their binding affinity in complex with the protein by combining sampling methods from Rosetta with a consensus metric using multiple protein-ligand scoring functions. We benchmarked the sampling/scoring approach and applied mPARCE using a known protease structure bound to a peptide substrate [31]. The main design protocol and auxiliary method to parameterize the NNAAs are available in the repository: https://github.com/rochoa85/mPARCE.

## Methods

### Parameterization of non-natural amino acids

A set of *α*- L- and D-NNAAs were chosen based on those detected in bound peptide structures available in the PDB. The BIOLIP database was used to download the most recent dataset of protein-peptide complexes from the PDB (accessed in November 2021), and only those peptides with NNAAs in their structures were further taken into account. The SMILES for each NNAA were obtained from the Chemical Component Dictionary (CCD) and the RDKit package [32] was used to calculate their molecular weights, filtering the amino acids with a molecular weight below 300 Daltons. The SMILES were used as input for the rdkit-to-params package (https://github.com/matteoferla/rdkit_to_params) to assign correct atom names to the NNAAs, and generate tripeptides with the motif G-X-G where X is the corresponding NNAA. Then each tripeptide was subjected to a customized script to generate the Rosetta parameters using Rosetta internal modules.

The parameterization script automatizes the generation of the input file (i.e. the structure of the NNAA surrounded by glycines) with correct atom names and in MDL MOL format. Additional flags to assign the backbone atoms and connection points are added into the input file, in order to be read by the *molfile_to_params_polymer.py*. This script is available in the demo folder of the Rosetta distribution [33]. A total of 90 parameterized NNAAs plus the 20 natural amino acids were included into the design protocol, and the parameters files are available in the mPARCE code repository to be located in the Rosetta installation path.

The selected NNAAs were clustered based on their physico-chemical properties, which were split into three categories: charge, hydrophobicity, and size. The RDKit package was used to calculate logP, charges and the isoelectric point for each NNAA capped with acetyl group and methylamine. Then, a set of thresholds were defined to assign a group category for each NNAA. Details of the thresholds are available in the Supplementary Note 1. For each category, three groups are available: hydrophobic, polar, and charged for hydrophobicity; neutral, positive and negative for charge; and small, medium and large for the size category. Based on these groups, the user can decide to include NNAAs having similar physico-chemical properties based on previous knowledge of the protein binding site, the chemical nature of specific peptide residues, or structure-activity relationship (SAR) information.

### Benchmark analysis

In order to validate the sampling/scoring approach proposed in mPARCE, a controlled benchmark study was conducted using two datasets of protein-peptide complexes. The first consisted of six PDB files with proteins bound to pairs of peptides reporting affinity differences of at least 100-fold, and with values at nanomolar range (nM). The second, and more challenging dataset, contains nine pairs of protein-peptide complexes with affinity differences lower than 100-fold at nanomolar range. The range of mutations in the peptides goes from one single mutation to multiple residues that modify a maximum of 70% of the total sequence. This large range allows for the evaluation of how the scoring can discriminate between very similar peptides to dissimilar cases, which is crucial to assess the impact of large modifications of the initial peptide sequence in the protocol. Details of the modified peptide sequences for the first dataset are reported in the Supplementary Tables 1, and for the second dataset in the Supplementary Table 2. The goal is to rank the best binding peptide (towards its protein target) per system.

For all pairs, we evaluated how many of six selected protein-ligand scoring functions agree with the experimental rank-ordering differences and thus correctly rank the bound peptides. For this protocol we used six scoring functions: DLigand2 [34], Vina [35], Cyscore [36], NNscore [37], a Rosetta score configured for docking [38], and the internal Rosetta score used during the relaxation phases [39]. In the case of NNscore, the negative of the predicted value was used to enable ranking the molecules similar to the other functions. Details of each scoring function are provided in the Supplementary Note 2.

Each complex was sampled and scored using the mPARCE approach (explained in “Design protocol”), using the last conformation of the relaxation. We evaluated if the sign of the difference between the predicted scores agrees with the experimental activity difference (∆∆*G*). This is, for each scoring function, we checked if a peptide compared to the other one increases or decreases the activity as a dichotomous response.

### Application using a protease-peptide complex

As an application of the design protocol, we selected a granzyme H protease (PDB id 3tjv) bound to a 9-mer peptide substrate. Two design runs were performed. For the first, we allowed random mutation of four arbitrary positions within the peptide (i.e. positions 2, 4, 6 and 8) using any of the parameterized NNAAs. For the second strategy, we modified the same positions but only allowed NNAAs with similar properties with regards to their hydrophobicity, charge, and size. Specifically, we allowed mutations to neutral, hydrophobic, and medium size amino acids (see Supplementary Note 1). During both design runs, we attempted a total of 100 mutations that were accepted when four or more scoring functions agreed on a favorable mutation. After achieving the number of attempts, a pool of accepted sequences was prioritized as candidates for further validations.

## Results

### Design protocol

#### Sampling and mutation

The mPARCE design protocol goal is to explore efficiently the sequence space through a stochastic search guided by the potential affinity between the protein and the bound modified peptide. To run the protocol, a 3D structure of a protein-peptide complex is required. The protein-peptide complex is protonated and subjected to sampling using the Backrub method from Rosetta [29]. A total of 20,000 trials are run using a kT of 1.2. These parameters were optimized in previous studies of sampling protein-peptide complexes [16]. Then a score is calculated using the last frame of the Backrub trajectory and with either a single scoring function or a consensus methodology explained in the next section. Then, the fixbb package is used to randomly mutate any position of the peptide by any of the NNAAs previously parameterized and included into Rosetta [40]. The mutated complex is relaxed with flexible side chains [41] and subjected to Backrub simulations using the same parameters. A new score is calculated and compared with the previous one. Based on the acceptance criterion, the modification is accepted or rejected, and the process is iterated for a selected number of times.

#### Scoring strategies

**Fig. 1:**
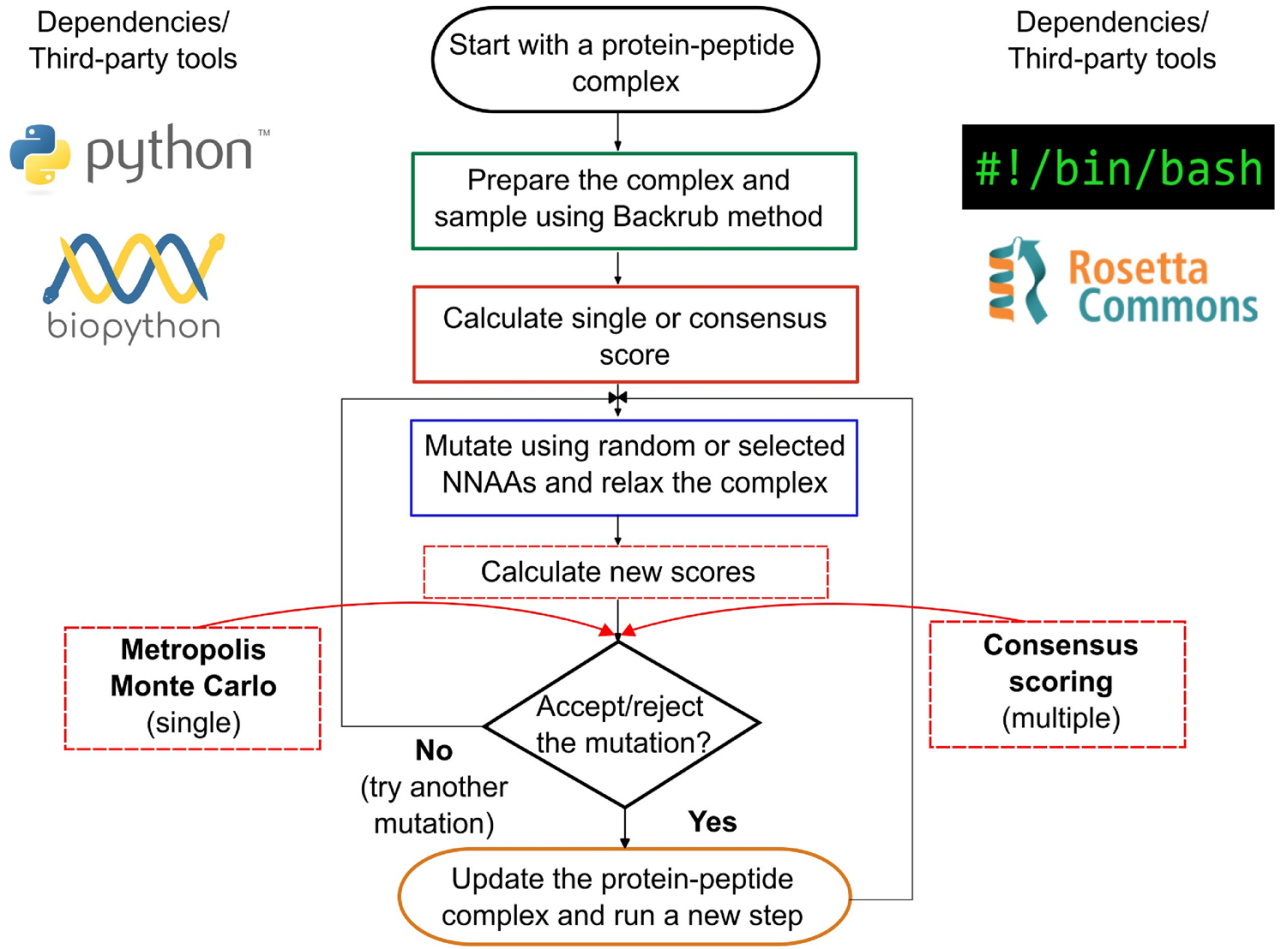
Design protocol. Schematic representation of the modified peptide design protocol (mPARCE) that optimizes the peptides following a stochastic methodology. It includes three main phases: a single-point mutation over a peptide chain, relaxation of the new protein-peptide complex, and the scoring of the new complex that allows the acceptance or rejection of the mutation. The scoring can be done using a single scoring function following a Metropolis-Hastings Monte Carlo strategy [42], or with a consensus scoring approach. The protocol is iterated to modify the peptide and improve its binding towards the target of reference

After sampling the mutated protein-peptide complexes, the last frame can be scored using a single scoring function or applying a consensus metric based on the selected set of scoring functions used for protein-ligand affinity predictions (see Methods). The mutation acceptance can be determined with two approaches. If a single scoring function is selected, the comparison is assessed by a Metropolis-Hastings Monte Carlo criterion [42] using an effective temperature between 1 and 10 (i.e., 1 is stricter to accept mutation if the difference score is not favorable, and 10 is more relaxed) [11]. If multiple scoring functions are selected, a consensus-based approach with the chosen N scoring functions is applied. In this case, if a particular number n of scoring functions agrees with negative scoring differences between the previous and mutated peptide, then the final consensus will accept the change and update the system [43]. The evolution of the peptide is iterated over a selected number of times to achieve better scores and explore the best candidates. A complete summary of the protocol is shown in Fig. 1.

### Benchmark outputs

**Fig. 2:**
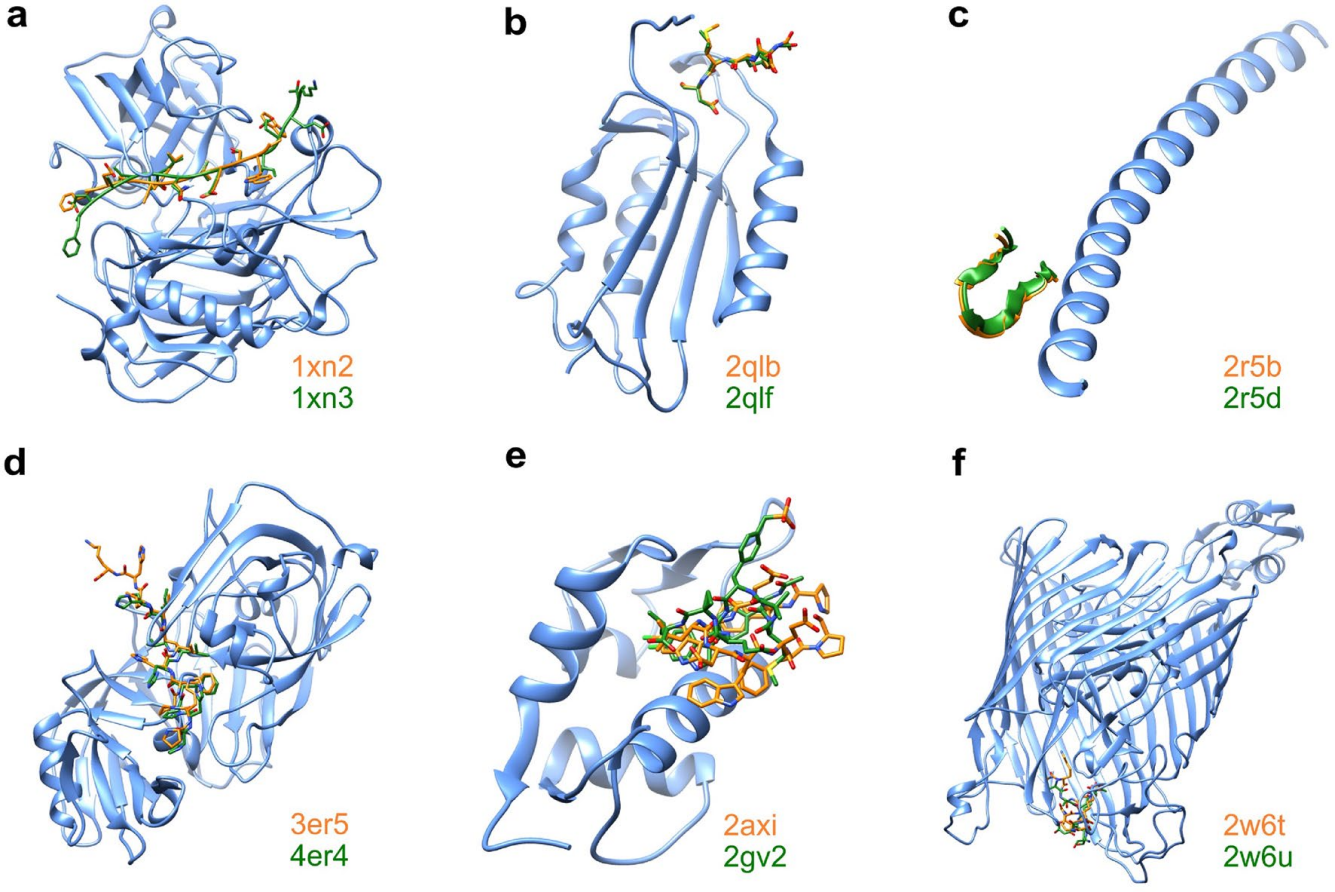
Benchmark systems of the first dataset. A total of six protein systems each bound to a pair of modified peptides. The protein is colored in blue, and the peptides in orange and green, with the corresponding PDB ids of the protein-peptide complexes. The systems are: **a** human beta-secretase from human (PDB ids 1xn2 and 1xn3) [44], **b** human caspase-7 (PDB ids 2qlb and 2qlf) [45], **c** HIV-1 gp41 N-trimer pocket region (PDB ids 2r5b and 2r5d) [46], **d** Endothiapepsin from *Cryphonectria parasitica* (PDB ids 3er5 and 4er4) [47], **e** human HDM2 (PDB ids 2axi and 2gv2) [48], and **f** FpvA from *Pseudomonas aeruginosa* (PDB ids 2w6t and 2w6u) [49]. All the complexes report K_d_ and IC_50_ values at nanomolar range (nM) (Table 1)

After running the Backrub trajectories with each PDB structure, six scoring functions were used to score the last frame. Then, we checked which scoring favored the same rank-ordering of the peptides as the experimental results. Based on that, we counted in which scenarios more than half (i.e., ≥ 4) of the scoring functions agree with the experimental ranking. This analysis was run for two datasets, one of them containing six pairs of protein-peptide complexes with 100-fold affinity differences. Details and structural representations of the six complexes are shown in Fig. 2.

The results for the first dataset are summarized in Table 1. Specific scoring values are given in Supplementary Table 3.

**Table 1:** Number of matches for the first benchmark dataset based on the number of scoring functions in agreement during the consensus ranking analysis for each pair of protein-peptide complexes

PDB id 1	Affinity 1 (nM)	PDB id 2	Affinity 2 (nM)	Matches
1xn2	0.03	1xn3	40	5
2qlf	1.4	2qlb	1300	3
2r5d	0.07	2r5b	2.5	3
2w6t	2.7	2w6u	10,000	5
2axi	5	2gv2	140	5
3er5	1	4er4	160	4

The PDB codes and the affinity values in nanomolar (nM) range are shown

In four of the six pairs, four or more scoring functions correctly predicted the affinity ranking. Based on our analysis, the consensus scheme should be able to discriminate between peptides differing in multiple amino acids (see Supplementary Table 1). We note that using the consensus scheme is beneficial given that the protein-ligand scoring functions can complement each other due to the limitations of predicting protein-peptide affinity scores [50].

We followed a similar analysis with a second dataset containing nine pairs of protein-peptide complexes but with lower affinity differences. Details of the included complexes are available in the Supplementary Note 3, and the main results are summarized in Table 2. We found that despite the challenging affinity differences, six of the nine complexes were able to surpass the defined threshold to rank each pair of peptides. Specific scoring values are given in Supplementary Table 4. Overall, these results allowed us to use the same scoring strategy to design modified peptide binders for a known protease-peptide complex to check the protocol performance.

**Table 2:** Number of matches for the second benchmark dataset based on the number of scoring functions in agreement during the consensus ranking analysis for each pair of protein-peptide complexes

PDB id 1	Affinity 1 (nM)	PDB id 2	Affinity 2 (nM)	Matches
2aoj	22,100	2aoi	96,700	4
2h5i	1.3	2h5j	12.4	1
6m9f	6	6m8y	415	4
2w16	0.1	2w78	2.7	3
3ove	270	3ov1	6250	4
1a1c	400	1a08	2400	4
1jyq	2	1zfp	26	3
4er2	0.5	2er9	40	5
5apr	17	4apr	200	4

The PDB codes and the affinity values in nanomolar (nM) range are shown

### Application using a granzyme H-peptide complex

**Fig. 3:**
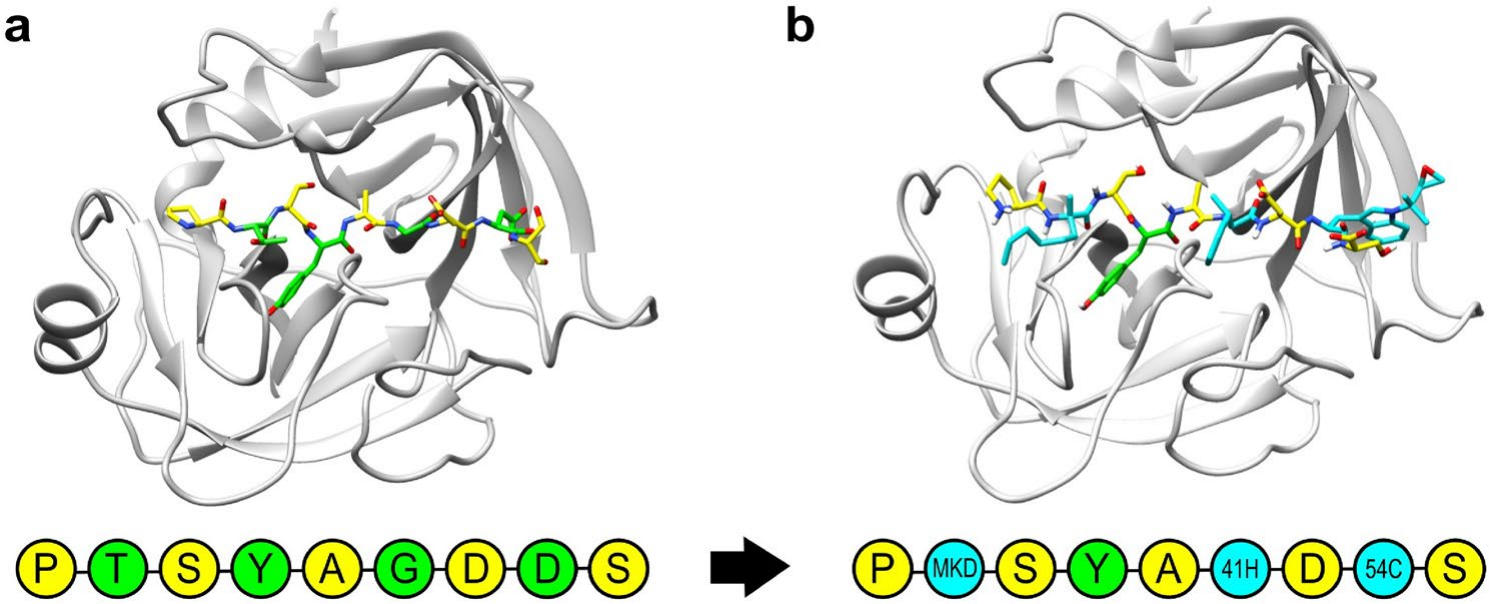
Application system. **a** Structure of the granzyme H (PDB id 3tjv) bound to the starting 9-mer peptide substrate. The positions selected to be modified are colored in green, and the remaining amino acids in yellow. **b** Final complex with the mutations accepted during the design protocol. The new NNAAs are colored in cyan, the position that remain unchanged in green, and the remaining positions in yellow. The final accepted sequence is shown using the PDB code names for the accepted NNAAs.

To test mPARCE we selected a well-characterized protease system (PDB id 3tjv) bound to a 9-mer peptide substrate. The peptide covers the cleavage binding site from position S4′ to S4, including the catalytic region between S1′ and S1 [51]. The peptide consists of 9 natural amino acids, and the goal was to allow changes in four positions, covering both the flanking and core amino acids close to the catalytic site. A structural view of the starting system is shown in Fig. 3a.

After attempting 100 mutations, a total of seven mutations were accepted, covering three of the four positions marked to be modified. The number of accepted sequences is associated with the consensus threshold (i.e., the larger the threshold, the stricter the acceptance criterion). In particular, position 8 was the most susceptible to be improved given its interaction with an exposed part of the protease binding site. Position 4 remained the same after multiple attempts to be changed, probably because the binding subpocket is very specific for tyrosine (Fig. 3b). In Supplementary Fig. 1, we show the conformation of that tyrosine in comparison with other attempted mutations. From a structural point of view, the tyrosine rotamer accommodates tightly in the available cavity, generating a set of interactions that none of the attempted substitutions were able to improve. Table 3 shows the progress of the design process through the iteration steps where the new sequences were accepted, including the specific mutation and the new mutated peptide sequence. The calculated scores of each step are reported in the Supplementary Table 5.

**Table 3:** Accepted peptide sequences obtained during the design run

Iteration	Mutation	Peptide sequence
Step 0	Original	PTSYAGDDS
Step 2	G-6-[ORN]	PTSYA[ORN]DDS
Step 14	[ORN]-6-[41 H]	PTSYA[41 H]DDS
Step 27	[ASP]-8-[G5G]	PTSYA[41 H]D[G5G]S
Step 33	[G5G]-8-[C1J]	PTSYA[41 H]D[C1J]S
Step 36	[C1J]-8-[KHB]	PTSYA[41 H]D[KHB]S
Step 40	T-2-[MKD]	P[MKD]SYA[41 H]D[KHB]S
Step 86	[KHB]-8-[54 C]	P[MKD]SYA[41 H]D[54 C]S

The iteration step and the mutation with the format: [old AA]-position-[new AA] is provided. The NNAAs are represented using the PDB 3-letter codes

**Fig. 4:**
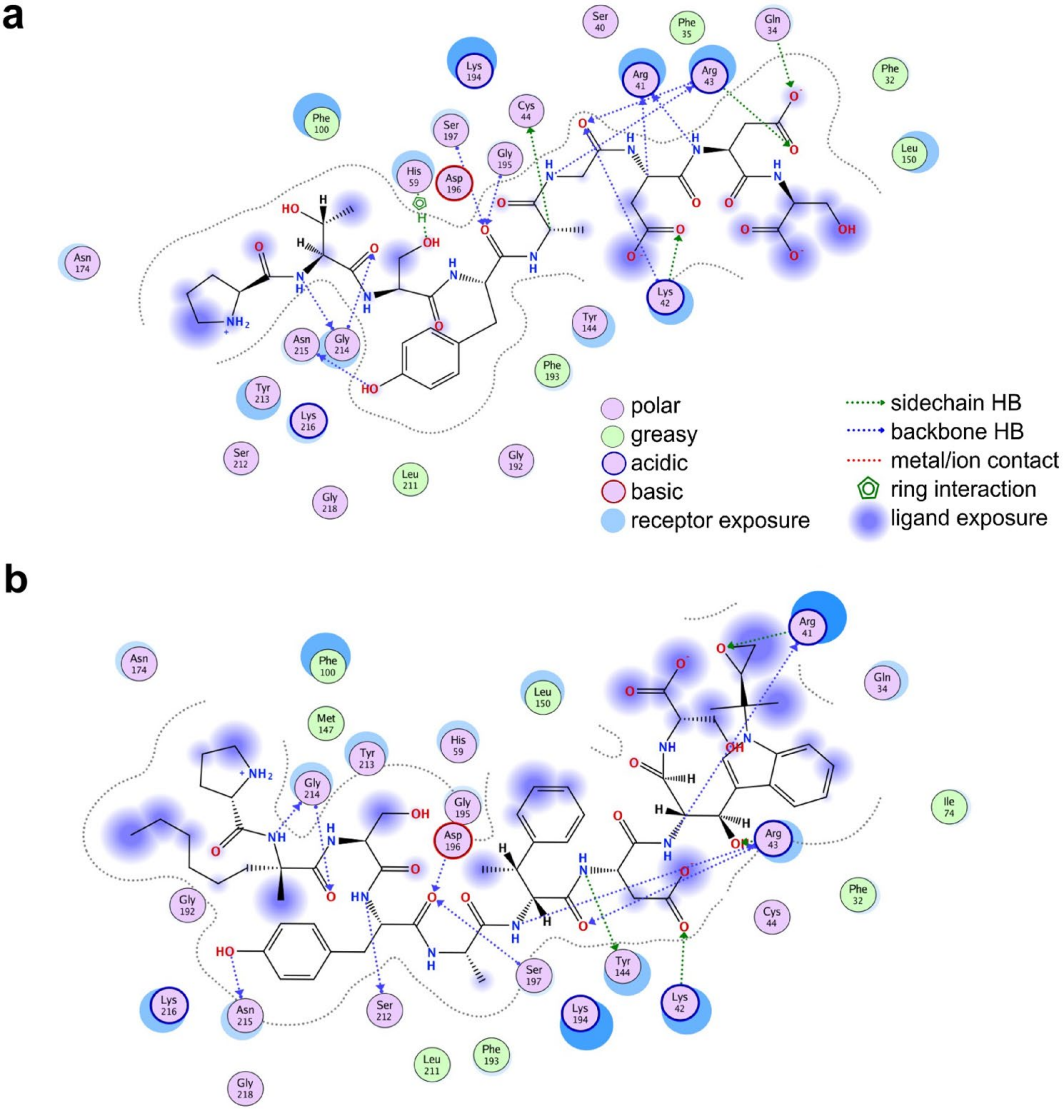
Peptide-protein interactions for the original and finally accepted peptide sequence. The chemical structure of the original peptide PTSYAGDDS (**a**) and the final modified sequence P[MKD]SYA[41 H]D[54 C]S (**b**) is shown. Protein residues are represented by circles, and main and side chain hydrogen bonds through dashed arrows. Receptor and ligand exposures as well as the physico-chemical nature of the residues are explained in the color legend. The diagrams were generated with the Molecular Operating Environment (MOE®) commercial software package

From a chemical composition perspective, hydrophobic NNAAs were accepted during the design such as MKD (i.e., (2 S)-2-amino-2-methyloctanoic acid) and modified versions of natural amino acids like 41 H, a methyl-L-phenylalanine and 54 C, a modified tryptophan. More information about the NNAAs can be found on the PDB using the 3-letter codes. However, a graphical representation of the protein-peptide interactions (depicting the peptide chemical structure for the original and final peptides) is shown in Fig. 4.

In general, the original and final modified peptides generate a similar number of backbone and side chain hydrogen bonds within the largely exposed binding site. However, the final modified peptide has the possibility to generate more hydrophobic interactions that can stabilize its binding pose. The latest behavior can be modified during the design by allowing the user to select just a subset of physico-chemically similar amino acids. To evaluate that, we ran a second design strategy but with a set of neutral, hydrophobic, and medium size amino acids from the original list. A list of the accepted sequences is shown in Supplementary Tables 6, and their calculated scores are in Supplementary Table 7. Similar to the previous scenario, the more exposed peptide positions were susceptible of being modified during the design process.

To validate the stability and rank predictions of the designed peptides from the first design strategy, we performed MD simulations of 500 ns using the initial sequence and the final modified peptide. The Amber package was used for both simulations, including the parameterization of the NNAAs. Details of the simulations are available in the Supplementary Note 4. We calculated the average score using the last half of the trajectory to rank the two peptides, which is in agreement with that predicted by mPARCE (see Supplementary Table 8). We found that the modified peptide tends to remain in the binding pocket in comparison to the initial sequence after 100 ns (Supplementary Fig. 2), which also suggests a better affinity of the designed peptide.

**Fig. 5:**
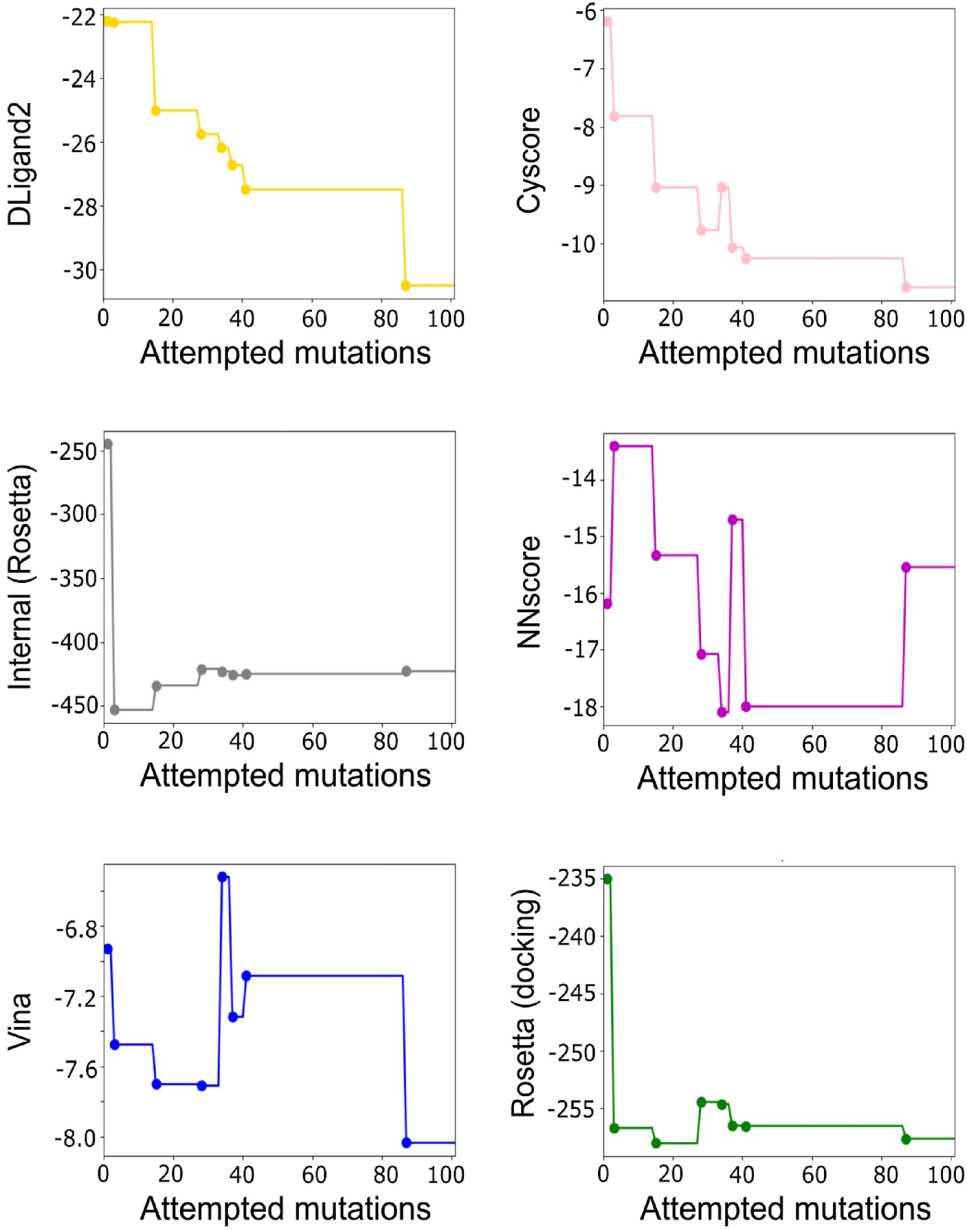
Evolution of the scoring functions using a consensus criterion. We used six scoring functions to calculate the consensus with a threshold of 4 after attempting 100 mutations. The dots in the curve represent the mutations that were accepted. The scoring functions used are DLigand2 (yellow), Cyscore (pink), Internal Rosetta score (gray), NNscore (magenta), Vina (blue) and Rosetta docking score (green)

Regarding the evolution of the scoring functions, we plotted the acceptance rate for the six scoring functions used in the design after 100 random mutation attempts (Fig. 5). We observed different behaviors for the scoring functions, with some showing convergence (i.e., Rosetta scores) and others with a decreasing tendency as in the case of Dligand2 or Cyscore. The performance can be optimized by attempting more mutations or changing the acceptance criteria. However, after comparing the accepted mutations between the six functions, the consensus metric can help overcome local minimization problems, as in the case of Vina between the 4th and 5th accepted sequences (Fig. 5). We also observe that NNscore finished the design run with a final sequence having a similar score with respect to the original molecule, but with previous sequences before the final mutation having better scores. This is expected given the stochastic nature of the sequence search that the consensus facilitates. A similar score tracking was performed for the second design run (see Supplementary Fig. 3).

### Code insights

The code, called mPARCE, is publicly available at: https://github.com/rochoa85/mPARCE/. The code was written in Python 3, with calls to third-party tools such as Biopython [52], Open Babel [53], Rosetta [27], and a set of protein-ligand scoring functions. The code was prepared and tested using the operating system Ubuntu 20.04. mPARCE on a single CPU core, and attempting 100 mutations, will require approximately 10 h. However, the user can update the code to call MPI-compiled versions of Rosetta to reduce the computational time using multiple cores. Another alternative to exploit parallelization is to launch multiple runs of the protocol simultaneously, using one core per run. In that way, multiple solutions can be obtained from each design by exploiting multi-processor acceleration. Regarding the computational resources to generate the NNAA parameters, our protocol can generate them in a few minutes for any monomer of interest using one single core.

The NNAA parameters and a csv with general information of each NNAA are available in the code repository. The chosen NNAAs contain chemical modifications only on the side chain, but the user has the option to include new NNAAs based on a correct parameterization of the structures in Rosetta. A script is available to automatically generate NNAAs parameters for Rosetta. The protocol requires a local Rosetta installation, and a set of instructions are provided to add the parameterized NNAAs into the program paths. These include the parameters and a master file with all the residue types read by the Rosetta functions. Instructions to do the full set up are provided in the code’s README file.

## Discussion

Here we describe a computational protocol to design modified peptides based on a starting protein-bound conformation and inspired by the PARCE protocol for peptide design [10]. The protocol allows a guided exploration of the sequence space through efficient Monte Carlo movers available in Rosetta. The design is achieved by single mutations on the binder chain, which are accepted or rejected based on a sampling/scoring hybrid approach. A benchmark of six peptide pairs to different protein targets showed that with the consensus scoring the most active peptide could be identified for most cases. A subsequent prospective study on a protease of therapeutic relevance yielded seven modified peptides, which can be prioritized for further studies.

The goal of the protocol is to provide a virtual screening approach to design modified analogs of bound peptides. To allow exploring a large chemical space by including NNAAs in the design protocol, we needed to implement efficient but less accurate tools to sample the peptide-bound conformations and their scoring. This is an alternative to more exhaustive computational methods such as molecular dynamics [9, 54], enhanced sampling [55] alchemical free energy perturbations [56], thermodynamic integration [57], among others. However, predicting affinity differences for highly flexible molecules such as peptides is still an active challenge, even for the more sophisticated methods [58]. Our hybrid Monte Carlo/scoring approach has shown promising results on datasets with peptides ranging from low to 100 times in the differences of the experimental affinities. Specifically, we have tested the proposed sampling/scoring approach with a set of proteases [51], and by ranking peptide binders of the MHC class II using the same sampling parameters of this study [16]. Based on these results, we expect that after attempting a considerable amount of mutations with our acceptance criteria, it can be possible to explore a larger number of sequences with potential better affinities. The final candidates can be re-ranked using more computationally demanding calculations.

One aspect about our protocol is the combination of diverse scoring functions, which have demonstrated to be useful for ranking peptide binders [16, 59] despite not being specific for predicting affinities of highly flexible molecules. Based on previous studies using the same consensus criteria, we found that accepting the mutations with three or four from the six scoring functions can be enough to explore efficiently the sequence space, avoiding overfitting or other statistical misleading effects [10, 43]. Using the consensus criterion is also a key differential factor to avoid relying exclusively on a single scoring function. However, during the consensus it is possible to observe underperforming functions given their dependency on the systems used to fit them. For example, we found that NNscore had a 60% of incorrect rankings for all the pairs of peptides used in the benchmark. An advantage of the consensus strategy is that it does not rely on all scoring functions performing well, and it overcomes these problematic cases to find better sequences based on optimizing a consensus score. In the case of getting more reliable scoring functions for at least certain protein-peptide complexes, running design projects using Metropolis Monte Carlo with a single scoring function is also a viable option that is included in the current code version.

Regarding the challenge of manipulating NNAA chemical structures, something relevant is to do a correct parameterization of them for the modelling and simulation steps [60]. One advantage of the Rosetta framework is the availability of tools to generate such parameters, in particular for new building blocks differing only in the amino acid side chain. With the expansion of the NNAAs chemical space that can be used in pharmacological applications [61], the user has the option to add novel entities in mPARCE by providing a 3D structure of the new monomer to generate the parameters. However, our protocol has been configured to do exclusively side chain modifications by conserving the original backbone. In the context of other type of amino acid modifications, or even more mimetic structures, our method is not suitable at the moment, but can be further adapted in order to guarantee a chemically correct substitution of the novel components. The latest can be complemented with the option of adding or deleting amino acids on the peptide chain to explore even bigger chemical spaces.

Finally, the code has been configured to facilitate its reproducibility under any computational infrastructure. The exhaustiveness is associated to the peptide sequence size and the number of attempted mutations. The mPARCE code is different from the original PARCE method in terms of the sampling method (Monte Carlo mover instead of MD) and the possibility to add NNAAs during the design. The dependencies can be easily installed, and we expect that mPARCE can become a valuable open option to design modified peptide binders of any protein target reporting resolved 3D-structures and characterized binding sites.

## Supplementary Information

Below is the link to the electronic supplementary material.

Supplementary file1 (PDF 1291 kb)

## Data Availability

The code, examples, and instructions to run the protocols are publicly available at: https://github.com/rochoa85/mPARCE.
