# Supplementary material for: Protocol for iterative optimization of modified peptides bound to protein targets
Source: J Comput Aided Mol Des. 2022 Oct 19;36(11):825–35. doi: 10.1007/s10822-022-00482-1 (PMC9640467; doi:10.1007/s10822-022-00482-1)
Supplement: Supplementary file 1 — Supplementary file1 (PDF 1291 kb) [file 10822_2022_482_MOESM1_ESM.pdf]

Supplementary Information: Protocol for iterative optimization of modified peptides bound to protein targets

Rodrigo Ochoa[1,2,*], Pilar Cossio[3,4], and Thomas Fox[2]

[1]*Biophysics of Tropical Diseases, Max Planck Tandem Group, University of Antioquia, 050010 Medellin, Colombia*

[2]*Medicinal Chemistry, Boehringer Ingelheim Pharma GmbH & Co KG, 88397 Biberach/Riss, Germany*

[3]*Center for Computational Mathematics, Flatiron Institute, New York 10010, United States of America*

[4]*Center for Computational Biology, Flatiron Institute, New York 10010, United States of America*

[*]*Corresponding author: rodrigo.ochoa@boehringer-ingelheim.com*

1 Supplementary Tables

Supplementary Table 1: List of PDB codes and peptide sequences used in the first dataset of the benchmark analysis. The non-natural amino acids (NNAAs) are surrounded by brackets.

PDB id	Peptide sequences
1xn2	WWSEVN[10L]AEF
1xn3	KTEEISEVN[STA]VAEF
2qlb	[ACE]ESM[ASJ]
2qlf	[ACE]DNL[ASJ]
2r5b	[DLY]G[DAL][DCY][DAS][DTY][DPR][DGL][DTR][DGN][DTR][DLE][DCY][DAL][DAL][NH2]
2r5d	[ACE][DLY]G[DAL][DCY][DAS][DTY][DPR][DGL][DTR][DGN][DTR][DLE][DCY][DAL][DAL][NH2]
2w6t	[DSN]KGKS[DSN]G[ORN]KS
2w6u	[DSN]A[AHO][ORN][DAS][AHO]S
2axi	PFEWLDWEF[DPR]
2gv2	[ACE]FMAFWE[1AC]L
3er5	PHPFH[STA]VIHK
4er4	PHPFH[LAV]IH

Supplementary Table 2: List of PDB codes and peptide sequences used in the second dataset of the benchmark analysis. The non-natural amino acids (NNAAs) are surrounded by brackets.

PDB id	Peptide sequences
2aoi	RPGN[FRD]LQSRP[NH2]
2aoj	VSFN[FRD]PQITAA[NH2]
2h5i	[ACE]DEV[ASJ]
2h5j	[ACE]DMQ[ASJ]
6m8y	[ACE]IP[PHL]
6m9f	[IVA]YL[TYE]
2w16	[DSN]R[DSN]KKKTT
2w78	[DSN]KGKKKS
3ov1	[ACT]YAN[NH2]
3ove	[ACT]Y[03E]N[NH2]
1a08	[ACE]YE[DIP]
1a1c	[ACE]YE[DIX]
1jyq	[MAZ]YYN
1zfp	[BE2]EYINQ[NH2]
2er9	[BOC]HPFH[STA]L
4er2	[IVA]VV[STA]A[STA]
4apr	PFH[STA]L
5apr	PFC[STA]LF[DHL]

Supplementary Table 3: Scores calculated for each protein-peptide complex selected from the PDB and included in the first dataset of the benchmark analysis.

PDB id	Rosetta (docking)	Vina	NNscore	Cyscore	Internal (Rosetta)	DLigand2
1xn2	-414.16	-3.93	-15.92	-4.23	209.11	-31.49
1xn3	-400.31	-2.41	-18.48	-2.81	323.61	-30.11
2qlb	-91.88	-1.39	-6.96	-0.62	339.07	-5.03
2qlf	-89.42	-1.52	-7.16	0.41	399.26	-6.27
2r5b	13.99	-1.95	-18.48	-0.98	718.03	-9.86
2r5d	-5.35	-1.66	-18.45	-0.60	423.31	-12.46
2w6t	-781.26	-3.08	-15.61	-1.91	66.89	-17.03
2w6u	-674.86	-0.44	-18.48	0.08	1359.70	-14.02
2axi	-43.67	-6.16	-9.79	-3.15	803.06	-24.34
2gv2	-27.76	-4.07	-13.66	-2.74	1172.79	-21.56
3er5	-284.94	-2.36	-18.46	-3.51	578.87	-29.52
4er4	-292.08	-2.34	-13.61	-5.68	671.65	-27.49

Supplementary Table 4: Scores calculated for each protein-peptide complex selected from the PDB and included in the second dataset of the benchmark analysis.

PDB id	Rosetta (docking)	Vina	NNscore	Cyscore	Internal (Rosetta)	DLigand2
2aoi	-182.658	-2.466	-18.484	-5.462	558.018	-26.994
2aoj	-193.741	-4.587	-16.977	-6.874	684.421	-29.112
2h5i	-209.973	-5.205	-8.828	-3.327	231.431	-10.64
2h5j	-220.993	-5.816	-17.173	-3.638	238.952	-12.106
6m8y	-142.634	-3.671	-6.723	-3.981	774.645	-8.475
6m9f	-150.981	-5.112	-4.663	-3.562	551.092	-10.338
2w16	-778.434	-2.562	-12.069	-1.654	-110.262	-15.696
2w78	-773.357	-3.161	-15.198	-2.166	75.2602	-13.184
3ov1	16.504	-4.079	-5.957	-1.071	1458.01	-7.431
3ove	99.015	-5.438	-6.685	-2.683	2500.92	-9.131
1a08	-33.939	-2.791	-3.968	-1.21	850.942	-6.901
1a1c	-79.93	-0.754	-14.67	-0.772	248.296	-7.547
1jyq	-88.592	-0.345	-18.441	-1.298	31.8014	-10.376
1zfp	-13.48	-3.411	-12.157	-2.371	1031.99	-12.018
2er9	-258.323	-0.975	-18.484	-4.344	1097.16	-19.36
4er2	-289.404	-1.822	-18.688	-2.925	752.593	-24.538
4apr	-291.663	-2.284	-18.125	-2.36	316.369	-20.111
5apr	-302.456	-2.057	-19.354	-4.78	457.455	-24.265

Supplementary Table 5: Scores for the accepted sequences during the design run using all possible NNAAs included in mPARCE. A total of 100 mutations were attempted.

Iteration	Vina	Dligand2	Rosetta (docking)	NNscore	Internal (Rosetta)	Cyscore
Step 0	-6.931	-22.197	-235.013	-16.19	-244.915	-6.189
Step 2	-7.476	-22.228	-256.752	-13.409	-453.105	-7.814
Step 14	-7.702	-25.006	-258.076	-15.335	-434.027	-9.037
Step 27	-7.71	-25.753	-254.487	-17.079	-420.957	-9.768
Step 33	-6.521	-26.182	-254.664	-18.109	-422.819	-9.04
Step 36	-7.318	-26.726	-256.542	-14.708	-425.881	-10.068
Step 40	-7.084	-27.492	-256.583	-18.009	-424.885	-10.254
Step 86	-8.035	-30.514	-257.692	-15.545	-422.783	-10.751

Supplementary Table 6: Accepted peptide sequences obtained during the design run using the filtered list of NNAA based on their properties. The iteration step and the mutation with the format: [old AA]-position-[new AA] is provided.

Iteration	Mutation	Peptide sequence
Step 0	Original	PTSYAGDDS
Step 4	T-2-[DIV]	P[DIV]SYAGDDS
Step 5	D-8-[NLE]	P[DIV]SYAGD[NLE]S
Step 7	G-6-[F7V]	P[DIV]SYA[F7V]D[NLE]S
Step 17	[DIV]-2-[NLE]	P[NLE]SYA[F7V]D[NLE]S
Step 25	[NLE]-2-C	PCSYA[F7V]D[NLE]S
Step 68	C-2-L	PLSYA[F7V]D[NLE]S

Supplementary Table 7: Scores for the accepted sequences during the design run using filtered NNAAs based on physico-chemical properties categories. A total of 100 mutations were attempted.

Iteration	Vina	Dligand2	Rosetta (docking)	NNscore	Internal (Rosetta)	Cyscore
Step 0	-7.707	-22.198	-244.77	-10.523	-258.756	-6.465
Step 4	-8.101	-22.068	-266.03	-18.48	-393.214	-6.963
Step 5	-7.973	-22.378	-267.181	-18.484	-391.385	-7.306
Step 7	-8.473	-23.376	-267.293	-15.642	-392.124	-8.367
Step 17	-8.151	-23.458	-267.858	-15.707	-396.04	-8.423
Step 25	-8.309	-23.374	-267.95	-15.930	-393.746	-8.528
Step 68	-8.468	-23.64	-268.835	-15.448	-395.936	-8.834

Supplementary Table 8: Average scores for the initial and final peptide based on scoring the last half of the MD trajectories.

Peptide	Vina	Dligand2	Rosetta (docking)	NNscore	Internal (Rosetta)	Cyscore
Initial	-3.834	-13.781	-213.447	-16.668	648.339	-3.175
Final	-7.202	-19.684	-259.398	-17.367	324.574	-6.653

2 Supplementary Figures

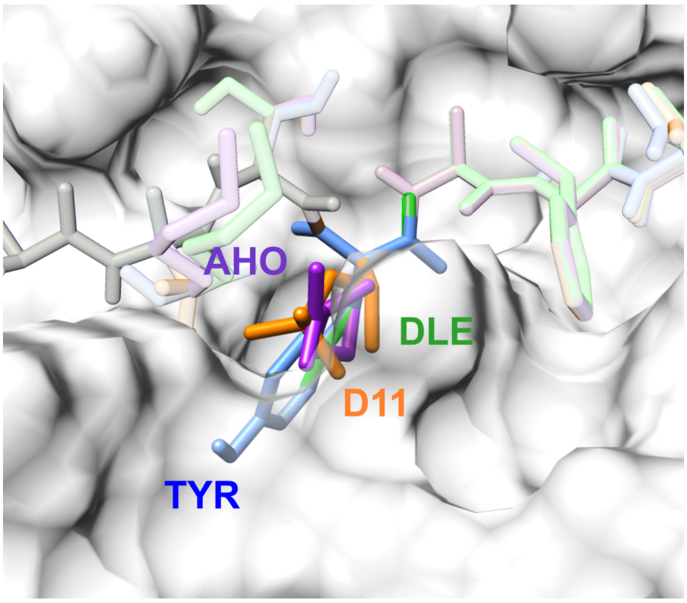

Supplementary Figure 1: Structural overlapping of different residues at the 4th position of the peptide. The original tyrosine (TYR) is represented in blue, and the rest are the attempted mutations AHO (purple), DLE (green) and D11 (orange). The protein cavity is colored light gray in transparency.

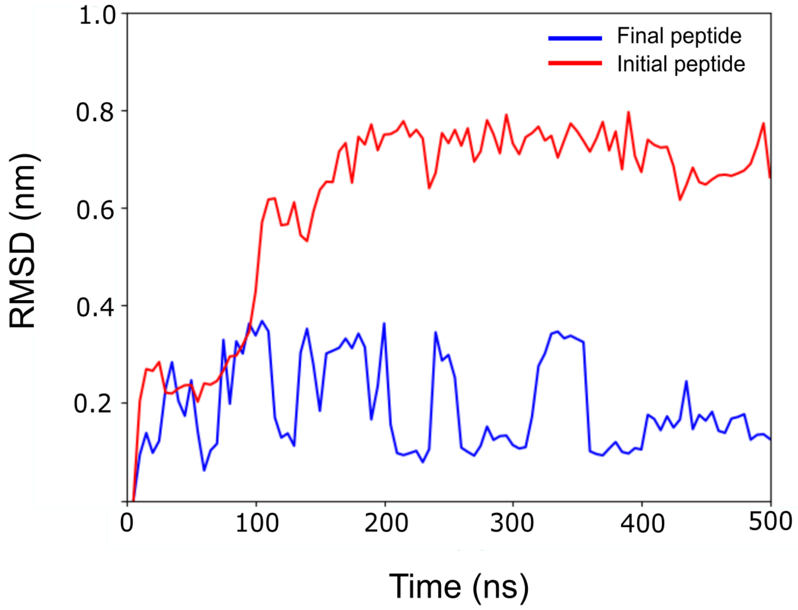

Supplementary Figure 2: RMSD of the initial peptide used for the design (red), and the final designed peptide of the random design strategy (blue). A total of 100 frames were obtained from MD simulations of 500 ns.

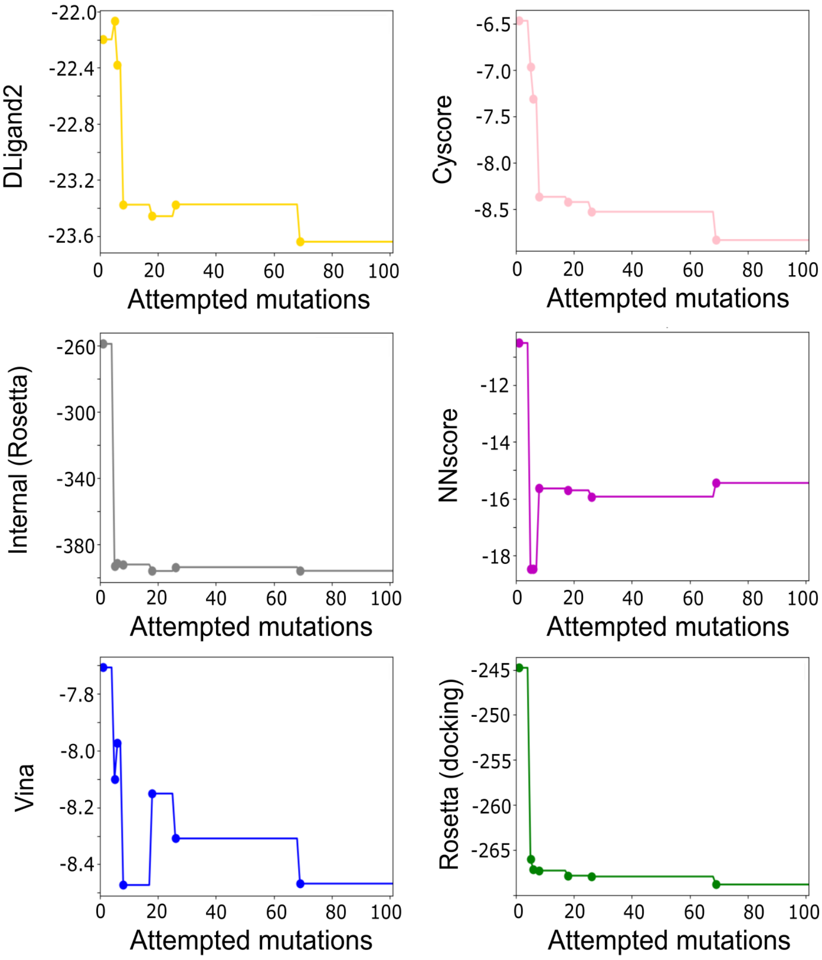

Supplementary Figure 3: Evolution of the scoring functions using a consensus criterion for the design run using property categories. We used six scoring functions to calculate the consensus with a threshold of 4 after attempting 100 mutations. The dots in the curve represent the mutations that were accepted. The scoring functions used are DLigand2 (yellow), Cyscore (pink), Internal Rosetta score (gray), NNscore (magenta), Vina (blue) and Rosetta docking score (green).

3 Supplementary Notes

3.1 Thresholds used to define the NNAAs categories

To classify the NNAAs a set of properties were calculated using 2D-representations of their chemical structures. Specifically, the molecular weight (MW) was calculated with the RDKit (https://rdkit.org/), and the logP, charges and isoelectric point (IP) with the MarvinSketch calculator from ChemAxon (https://chemaxon.com/products/marvin).

To generate the groups within the size category, the weights were defined as: small for MW lower than 110 daltons, medium for MW between 110 and 135 daltons, and large for MW greater than 135 daltons. The charge groups were: negative for charges lower than -0.1, positive for charges greater than 0.1, and neutral for the ones between -0.1 and 0.1. Finally the hydrophobicity groups were defined as: charged if the NNAA is defined as positive or negative based on the previous category, polar if the logP is lower than -3 and the IP lower than 5.75 or if the logP is greater than -3 and the IP lower than 5.55, and hydrophobic for the rest of the logP and IP value intervals. The thresholds were fitted based on the categories assigned to the natural amino acids.

3.2 Scoring function details

- Rosetta (version 2016.32): Statistical method composed of multiple energy terms optimized for different kinds of interactions, including protein-protein interactions. The all-atom terms describe the individual residue environments and frequent residue-pair interactions obtained from the Protein Data Bank (PDB). Additional terms account for packing of van der Waals spheres, hydrogen bonding and secondary structure elements. The score used was also optimized for docking calculations, and two versions are used in the consensus schema applied within the protocol [1, 2].

- Vina (version 1.1.2): Program derived from AutoDock, used mainly for docking of small molecules and small peptides against protein structures. The scoring function uses a semi-empirical approach, with a conformation-dependent part that includes the terms about repulsion, hydrophobic interactions, hydrogen bonding, and number of rotations. Despite that it is used with smaller ligands, its generalization lets us apply it for larger oligopeptides in bound conformations [3].

- Cyscore (Version 2014): Cyscore is an empirical scoring function for accurate protein-ligand binding affinity prediction. It is composed of hydrophobic free energy, van der Waals interaction energy, hydrogen-bond energy and the ligand's entropy. To improve the prediction accuracy, a curvature weighted surface area model was developed for the hydrophobic free energy calculation [4].

- NNScore (Version 2.0): Contrary to the previous methods, NNscore is a neural-network-based scoring function optimized to predict protein-ligand affinities. In this version the program includes multiple features and binding characteristics to improve the prediction of energies for docking campaigns [5].

- DLigand2 (Version 2.0): This scoring function is an improved knowledge-based potential previously used to score not only small molecules, but oligonucleotides, peptides and carbohydrates. [6].

3.3 Benchmark systems of the second dataset

A total of nine protein systems each bound to a pair of modified peptides were included for the analysis. The systems are: HIV-1 protease (PDB ids 2aoi and 2aoj) [7], human caspase-3 (PDB ids 2h5i and 2h5j) [8], serine-carboxyl proteinase from *Pseudomonas sp. 101* (PDB ids 6m8y and 6m9f) [9], FpvA from *Pseudomonas aeruginosa* (PDB ids 2w6t and 2w6u) [10], Grb2 SH2 domain from human (PDB ids 3ov1 and 3ove) [11], C-SRC SH2 domain from human (PDB ids 1a08 and 1a1c) [12], Grb2 SH2 domain from human (PDB ids 1jyq and 1zfp) [13], Aspartic proteinase from *Cryphonectria parasitica* (PDB ids 2er9 and 4er2) [14], and Rhizopuspepsin from *Rhizopus microsporus* (PDB ids 4apr and 5apr) [15]. All the complexes report Kd and IC50 values at nanomolar range (nM) (Table 2).

3.4 MD simulation

The system was solvated with the TIP3P water model [16] in a truncated cubic box, extending 10 Å from the protein. A physiological salt concentration of 0.15 M was used, employing Na^+ an Cl^- ions. Finally, hydrogens were added using the Tleap module of the Amber simulation suite [17]. The simulation was performed using the PMEMD cuda module of the Amber simulation suite, and consisted on: an initial 1 picosecond (ps) run with a 0.01 femtosecond (fs) timestep to eliminate bad contacts, followed by an energy minimization. The system was then equilibrated for 200 ps at constant temperature and pressure with weak restraints on the CA atoms of the protein (1 kcal/mol/Å^2). The Langevin thermostat was used with a collision frequency of 2.0 ps^{-1} [18], the SHAKE algorithm was used to constrain bonds, allowing a 2 fs timestep, and an 8.0 Å cutoff was used for non-bonded interactions. Finally, a 500 nanoseconds (ns) production simulation was performed under the NPT conditions described above.

References

[1] S. T. Smith and J. Meiler, "Assessing multiple score functions in rosetta for drug discovery," *PloS One*, vol. 15, no. 10, p. e0240450, 2020.

[2] R. F. Alford, A. Leaver-Fay, J. R. Jeliazkov, M. J. O?Meara, F. P. DiMaio, H. Park, M. V. Shapovalov, P. D. Renfrew, V. K. Mulligan, K. Kappel, J. W. Labonte, M. S. Pacella, R. Bonneau, P. Bradley, R. L. Dunbrack, R. Das, D. Baker, B. Kuhlman, T. Kortemme, and J. J. Gray, "The Rosetta All-Atom Energy Function for Macromolecular Modeling and Design," *Journal of Chemical Theory and Computation*, vol. 13, pp. 3031–3048, jun 2017.

[3] O. Trott and A. J. Olson, "AutoDock Vina: Improving the Speed and Accuracy of Docking with a New Scoring Function, Efficient Optimization, and Multithreading," *Journal of Computational Chemistry*, vol. 31, no. 16, pp. 455–461, 2009.

[4] Y. Cao and L. Li, "Improved protein–ligand binding affinity prediction by using a curvature-dependent surface-area model," *Bioinformatics*, vol. 30, no. 12, pp. 1674–1680, 2014.

[5] J. D. Durrant and J. A. McCammon, "Nnscore 2.0: a neural-network receptor–ligand scoring function," *Journal of Chemical Information and Modeling*, vol. 51, no. 11, pp. 2897–2903, 2011.

[6] P. Chen, Y. Ke, Y. Lu, Y. Du, J. Li, H. Yan, H. Zhao, Y. Zhou, and Y. Yang, "Dligand2: an improved knowledge-based energy function for protein–ligand interactions using the distance-scaled, finite, ideal-gas reference state," *Journal of Cheminformatics*, vol. 11, 08 2019.

[7] Y. Tie, P. I. Boross, Y.-F. Wang, L. Gaddis, F. Liu, X. Chen, J. Tozser, R. W. Harrison, and I. T. Weber, "Molecular basis for substrate recognition and drug resistance from 1.1 to 1.6 å resolution crystal structures of hiv-1 protease mutants with substrate analogs," *The FEBS journal*, vol. 272, no. 20, pp. 5265–5277, 2005.

[8] B. Fang, P. I. Boross, J. Tozser, and I. T. Weber, "Structural and kinetic analysis of caspase-3 reveals role for s5 binding site in substrate recognition," *Journal of molecular biology*, vol. 360, no. 3, pp. 654–666, 2006.

[9] A. Wlodawer, M. Li, A. Gustchina, Z. Dauter, K. Uchida, H. Oyama, N. E. Goldfarb, B. M. Dunn, and K. Oda, "Inhibitor complexes of the pseudomonas serine-carboxyl proteinase," *Biochemistry*, vol. 40, no. 51, pp. 15602–15611, 2001.

[10] J. Greenwald, M. Nader, H. Celia, C. Gruffaz, V. Geoffroy, J.-M. Meyer, I. J. Schalk, and F. Pattus, "Fpva bound to non-cognate pyoverdines: molecular basis of siderophore recognition by an iron transporter," *Molecular microbiology*, vol. 72, no. 5, pp. 1246–1259, 2009.

[11] J. M. Myslinski, J. E. DeLorbe, J. H. Clements, and S. F. Martin, "Protein–ligand interactions: thermodynamic effects associated with increasing nonpolar surface area," *Journal of the American Chemical Society*, vol. 133, no. 46, pp. 18518–18521, 2011.

[12] P. S. Charifson, L. M. Shewchuk, W. Rocque, C. W. Hummel, S. R. Jordan, C. Mohr, G. J. Pacofsky, M. R. Peel, M. Rodriguez, D. D. Sternbach, *et al.*, "Peptide ligands of pp60c-src sh2 domains: a thermodynamic and structural study," *Biochemistry*, vol. 36, no. 21, pp. 6283–6293, 1997.

[13] P. Nioche, W.-Q. Liu, I. Broutin, F. Charbonnier, M.-T. Latreille, M. Vidal, B. Roques, C. Garbay, and A. Ducruix, "Crystal structures of the sh2 domain of grb2: highlight on the binding of a new high-affinity inhibitor," *Journal of molecular biology*, vol. 315, no. 5, pp. 1167–1177, 2002.

[14] J. Cooper, S. Foundling, T. Blundell, J. Boger, R. Jupp, and J. Kay, "X-ray studies of aspartic proteinase-statine inhibitor complexes," *Biochemistry*, vol. 28, no. 21, pp. 8596–8603, 1989.

[15] K. Suguna, E. A. Padlan, R. Bott, J. Boger, K. D. Parris, and D. R. Davies, "Structures of complexes of rhizopuspepsin with pepstatin and other statine-containing inhibitors," *Proteins: Structure, Function, and Bioinformatics*, vol. 13, no. 3, pp. 195–205, 1992.

[16] W. L. Jorgensen, J. Chandrasekhar, J. D. Madura, R. W. Impey, and M. L. Klein, "Comparison of simple potential functions for simulating liquid water," *Journal of Chemical Physics*, 1983.

[17] D. Case, R. Betz, D. Cerutti, T. Cheatham, III, T. Darden, R. Duke, T. Giese, H. Gohlke, A. Goetz, N. Homeyer, S. Izadi, P. Janowski, J. Kaus, A. Kovalenko, T. Lee, S. LeGrand, P. Li, C. Lin, T. Luchko, R. Luo, B. Madej, D. Mermelstein, K. Merz, G. Monard, H. Nguyen, H. Nguyen, I. Omelyan, A. Onufriev, D. Roe, A. Roitberg, C. Sagui, C. Simmerling, W. Botello-Smith, J. Swails, R. Walker, J. Wang, R. Wolf, X. Wu, X. L., and P. Kollman, "Amber 2016," *University of California, San Francisco*, 2016.

[18] R. L. Davidchack, R. Handel, and M. V. Tretyakov, "Langevin thermostat for rigid body dynamics," *Journal of Chemical Physics*, 2009.
